# Prevalence of type 1 diabetes mellitus in Karnal district, Haryana state, India

**DOI:** 10.1186/1758-5996-2-14

**Published:** 2010-03-09

**Authors:** Sanjay Kalra, Bharti Kalra, Amit Sharma

**Affiliations:** 1 Bharti Hospital & B.R.I.D.E., Karnal, 132001, India

## Abstract

**Background:**

Little work has been done on the prevalence of type 1 diabetes in north India. This paper reports the prevalence of type 1 diabetes in Karnal district of Haryana state, India.

**Materials and methods:**

Prevalence of type 1 diabetes was assessed by a hospital-based registry and by analysis of data contributed by chemists and other physicians.

**Results:**

The overall prevalence of type 1 diabetes in Karnal district is 10.20/100,000 population, with a higher prevalence in urban (26.6/100,000) as compared to rural areas (4.27/100,000). Karnal city, with a population of 222017, has a relatively high prevalence of type 1 diabetes (31.9/100,000). The prevalence in men is higher (11.56/100,000) than in women (8.6/100,000).

In the 5 to 16 years age group, the prevalence is 22.22/100,000, while in the 0-5 years age group, prevalence is 3.82/100,000.

**Conclusions:**

This report highlights the urban-rural and male-female gradient in the prevalence of type 1 diabetes in Karnal, north India.

## Introduction

While extensive work has been done to determine and document the increasing incidence of type 2 diabetes worldwide, comparatively less attention has focused on the prevalence of type 1 diabetes, especially in the developing world. The prevalence has been studied by various methods, including surveys, national or central registries, school records and hospital records, in various age groups [1]. It varies from 0.09 per 1000 in China, to 0.83-2.23 in Scandinavia and 3.40 in United Kingdom [1].

Incidence studies are available from many countries and reveal rates ranging from 36.5/100,000 in Finland and 36.6/100,000 in Sardinia (Italy) [2] to 0.1-4.6/100,000 in China and 0.4/100,000 in Thailand [3,4]. The incidence is low in all Asian countries studied viz. Japan, China, Hong Kong, Pakistan and Thailand [3,4].

In India, the Karnataka type 1 diabetes registry reports an incidence of 3.7/100,000 in boys and 4.0/100,000 in girls, over 13 years of data collection [5]. The incidence of type 1 diabetes has been reported as 10.5/100,000 in a population based study carried out in 1996 in urban Chennai [6].

Previous studies have revealed a prevalence rate of 1.6 to 10.1 per 100 000 population in India [7,8]. This paper reviews the prevalence of type 1 diabetes mellitus in Karnal, a district in Haryana state, northern India.

## Methods

This study was planned to assess the prevalence of type 1 diabetes in Karnal district. Karnal is a geographically small district, with one large city, Karnal, five other urban centres or municipal committees (Gharaunda, Assandh, Nilokheri Taraori and Indri) and a rural hinterland of 434 villages [9].

The district has a large civil hospital, 5 community health centers and 26 primary health centers, 12 dispensaries and 141 subcentres [9]. Most pediatricians and internists do not manage complicated type 1 diabetes, preferring to refer them to specialized centers.

Endocrine care is provided by the lone endocrinology centre, based in Karnal, which till recently, had the only endocrine specialist in the state. Insulin is available at many chemist outlets, but the limited number of wholesalers of the drug makes it easy to track prescriptions.

A registry of diabetes patients being maintained at the endocrine centre allowed one to identify most type 1 diabetes patients of the district. Type 1 diabetes was defined by clinical parameters [10], including absolute need for insulin, young age of onset and history of ketosis, for the purpose of this study. Absolute need for insulin was defined as a clinically diagnosed requirement for insulin therapy, with or without history of non-response to oral hypoglycaemic agents. Young age of onset was taken as onset of diabetes prior to 18 years of age. History of ketosis, ketonuria or diabetic ketoacidosis was elicited from clinical records. Autoimmune markers were not checked in routine practice, due to financial constraints.

This study was performed over a period of three months, from June to August 2008. Data of all type 1 diabetes patients attending the only endocrine OPD in the region was analysed and screened for patients living in the district. All chemists in insulin business were contacted through wholesalers of the drug and requested about details of children and young adults purchasing insulin. Similarly all 18 pediatricians and 22 internists working at other urban centres within the district were requested for details of patients being treated by them. All names and addresses were cross checked to avoid duplication. Patients from other districts and states were excluded, as well as those who had given addresses of relatives residing in Karnal City.

Population data were taken from the 2001 national census of India [9,11]. This counted a total of 1,274,183 people in the district, including 337,842 urban dwellers and 936,341 villagers. The 337,842 urbanites included 222,017 inhabitants of the district headquarters, Karnal city [9]. The gender ratio in the district was 864.6 females for 1000 males (590,815 women; 683,368 men), which is typical of northwest India. The gender imbalance was more pronounced in the 0-6 year age group, which contained only 808 girls/1000/boys.

The age distribution revealed a young population: 130,815 were aged 0-4 years, 317,912 were 5-14 years old, 729,685 were 15-59 years old and 95,771 aged above 60 years [9].

## Results

The survey revealed 79 males and 51 females with type 1 diabetes in the district. Of these 54 men and 36 women were from urban areas, while 25 men and 15 women were from a rural background.

The overall prevalence of type 1 diabetes in Karnal district was 10.20/100,000 population (130/1,274,183), with a higher prevalence in urban areas: 26.6/100,000(90/337,842) as compared to rural areas: 4.27/100,000 (40/936,341). Karnal City, with a population of 222,017, showed a higher prevalence of type 1 Diabetes: 31.9/100,000 (total 71 patients), as compared to other urban and rural areas. (Table 1)

**Table 1:** prevalence (per 100 000) of type 1 diabetes in Karnal

Age group (years)	Karnal district	rural	Karnal City
total	10.2	4.27	31.9 (26.6 urban)

5-14 years	24.22	-	-

0-6 years	3.82	0.00	19.9

The prevalence in men of the district was much higher (11.56/100,000 or 79/683,368) than in women (8.6/100,000 or 51/590,815).

Prevalence of type 1 diabetes in Karnal District was 24.22/100,000 (77/317,912) in the 5 to 14 years age group, and slightly lower: 18.27/100,000 (82/448727) in the 0 to 14 years group.

In the toddler (0 to 6 years) age group, type 1 diabetes prevalence was 21.2/100,000 (3/14,119) and 18.2/100,000 (2/10,931) respectively, in boys and girls of Karnal City. The prevalence of diabetes in the 0 to 6 years age group in the district was only 3.82/10,0000 (5/130,815). This occurred because no rural children in this age group were registered with type 1 diabetes (Table 1)

## Discussion

This prevalence study uses a simple method of data collection, focusing on an out patient registry and data collected from all stakeholders viz, physicians, pediatricians, wholesalers and retailers of insulin. This method is useful in areas where the number of healthcare providers and drug providers is limited, and can be replicated in many parts of the world.

The data reveals a higher urban prevalence of type 1 diabetes in India than thought previously.

This finding may be explained by a higher penetration of endocrine awareness and type 1 diabetes care in this district, as it has a dedicated endocrine centre for nearly a decade. It may also be due to under reporting from other parts of the country or may reflect an actual increase in incidence over the past decade.

The type 1 diabetes prevalence in Karnal exhibits a marked urban: rural and male: female gradient. The higher incidence of diabetes in urban population has been reported by other workers, but the steep male: female difference seems to be due to social and cultural factors, discussed earlier by the authors [12]. Girl children in India suffer from discrimination, both overt and covert, and are less likely to get adequate medical care than their male peers [13].

This study has a few strengths as compared to previous reports from the region. The prevalence of type 1 diabetes has been calculated for the whole population, rather than an arbitrarily chosen paediatric age group, to reflect the increasing occurrence of the condition in 'elder' people.

Though the primary source of data has been an OPD- based registry, it has been buttressed by inputs from other stakeholders such as physicians, paediatricians and insulin wholesalers as well as retailers. Thorough cross checking of data has been done to ensure that no duplication occurred.

The methodology used may have missed a few type 1 diabetes patients who have been taking treatment, and buying insulin from outside the district. Even then, this methodology cannot overestimate the magnitude of type 1 diabetes.

The figures reported may be extrapolated to the country. However, the prevalence of type 1 diabetes is said to increase with increasing latitude, i.e. with increasing distance from the equator [14]. Hence, the data may be justifiably used only for the northern regions of the south Asian subcontinent.

In spite of these potential limitations, the knowledge gained from this work represents an important addition to our understanding of the prevalence of type 1 diabetes in India.

The data has important implications for health policy makers, service providers and all stakeholders in diabetes.

The rural: urban population ratio of Karnal District is similar to that of the rest of India. The level of industrialization is average, by national standards, though the district is more affluent than many others. However, its' female: male ratio, though typical of north-west India, is less than that found in other states. In spite of this limitation, however, it highlights the fact that there is a large population of type 1 diabetes in India (population = 1,116,079,217) [15]. This issue cannot be ignored while planning any public health strategy.

## Conclusion

This paper adds important information about the epidemiology of type 1 diabetes in India.

This work highlights the relatively high incidence of type 1 diabetes in north India, with a high male: female gender ratio and high urban: rural gradient, using a simple, economical methodology. This methodology may be appropriate for assessing the prevalence of diabetes in many developing nations.

## Conflict of interests

The authors declare that they have no competing interests.

## Authors' contributions

SK conceived the study. All authors contributed to paper writing and data search.

All authors read and approved the final manuscript.

## References

[B1] Harris MI Diabetes in America 1995 2 Bethesda, MD, National Institutes of Health

[B2] Karvonen M Vik-Kajander M Moltchhanova E Incidence of childhood type 1 diabetes worldwide. Diabetes Mondiale (DiaMond) project group Diabetes Care 2000 23 1516 1526 10.2337/diacare.23.10.1516 11023146

[B3] EURODIAB ACE study group Variation and trends in incidence of childhood year diabetes in Europe Lancet 2000 355 873 876 10.1016/S0140-6736(99)07125-1 10752702

[B4] Unachak K Tuchinda C Incidence of type 1 diabetes in children under 15 years in northern Thailand, from 1991 to 1997 J Med Assoc Thai 2001 84 923 928 11759972

[B5] Prasanna Kumar KM Krishna P Reddy SC Gurrappa M Aravind SR Munichoodappa C Incidence of type 1 diabetes mellitus and associated complications among children and young adults: results from Karnataka Diabetes Registry 1995-2008 J Indian Med Assoc 2008 106 708 711 19368094

[B6] Ramachandran A Snehalatha C Krishnaswamy CV The Incidence of IDDM in children in urban population in southern India. Madras South India Diabetes Res Clin Pract 1996 34 79 82 10.1016/S0168-8227(96)01338-1 9031809

[B7] Ramachandran A Snehalatha C Sasikala R Satyavani K Vijay V Vascular complications in Young Asian Indian patients with type 1 diabetes mellitus Diab Res Clin Pract 2000 48 51 56 10.1016/S0168-8227(99)00134-5 10704700

[B8] Ramachandran A Snehalatha C Khader O Joseph TA Visvanathan M Prevalence of childhood diabetes in urban population in South India Diab Res Clin Pract 1992 17 227 231 10.1016/0168-8227(92)90098-C 1425162

[B9] Karnal district population statistics http://www.karnal.nic.in Accessed on 5 July 2009.

[B10] AMERICAN DIABETES ASSOCIATION Diabetes mellitus Diabetes Care 2010 33 Suppl 1 S62 S69 20042775 10.2337/dc10-S062 PMC2797383

[B11] India Census statistics http://www.censusindia.gov.in/Dist_File/datasheet-0606.pdf Accessed on 5 July 2009

[B12] Kalra S Promoting opportunities, fighting against isolation in India Diabetes Voice 2008 53 Spl 46 48

[B13] Kalra B Kalra S Sharma A Social stigma and discrimination: a care crisis for young women with diabetes in India Diabetes Voice 2009 Spl 37 39

[B14] Karvonen M Tuomiletho J Libman I A review of the recent epidemiological data on incidence of type 1 (insulin-dependent) diabetes mellitus worldwide Diabetologia 1993 36 883 892 10.1007/BF02374468 8243865

[B15] India population statistics http://en.wikipedia.org/wiki/Demographics_of_india Accessed on 5 July 2009.

